# Larg-scale multimodal fMRI dataset of visual and haptic 3D object category learning

**DOI:** 10.1038/s41597-026-07814-y

**Published:** 2026-07-16

**Authors:** Sepideh Tabrik, Sama Rahnemayan, Martin Tegenthoff, Mehdi Behroozi

**Affiliations:** 1 Department of Neurology, BG-University Hospital Bergmannsheil, Ruhr University Bochum, 44789 Bochum, Germany; 2 Department of Information Technology, University of Applied Sciences and Arts Dortmund, Dortmund, Germany; 3 Department of Neurosurgery, Medical University of Vienna, Vienna, Austria; 4 High Field MR Centre, Department of Biomedical Imaging and Image-guided Therapy, Medical University of Vienna, Vienna, Austria; 5 Institute of Cognitive Neuroscience, Department of Biopsychology, Faculty of Psychology, Ruhr University Bochum, 44780 Bochum, Germany; 6 Cognitive Neurobiology, Research Center “One Health” Ruhr, University Alliance Ruhr, Faculty of Biology and Biotechnology, Ruhr-University Bochum, Universitätsstraße 150, 44801 Bochum, Germany

**Keywords:** Cognitive neuroscience, Short-term memory

## Abstract

How do humans recognize objects in their environment using either vision or touch, even when only one sense is available? Humans routinely integrate visual and haptic sensory systems for object recognition, yet the mechanisms behind cross-modal transfer of category information are not fully understood. In this study, we collected behavioral and fMRI data from 134 participants. Fifty participants (25 per group; 13 females each) performed a similarity rating task with novel 3D objects (“digital embryos”) using either visual or haptic modalities. The remaining 84 participants completed a 3D object category learning experiment in unimodal (Visual-Visual: n = 19, 11 females; Haptic-Haptic: n = 22, 10 females) or cross-modal (Visual-Haptic: n = 22, 11 females; Haptic-Visual: n = 21, 10 females) conditions. Alongside task-based fMRI, we acquired resting-state fMRI data at four time points: pre-training, post-training, pre-test, and post-test. High-resolution T1-weighted images were also obtained for each MRI session. These data support investigations into visual and haptic representations of the environment, representational similarity across modalities, cross-modal transfer of category information for object recognition, and neural plasticity in brain networks.

## Background & Summary

Picture this: You reach into your purse and, just by touch, locate your office keys among other items. Or sometimes, you find them only with a quick glance without touching them. Ever wonder why you can identify objects using either your sense of touch or vision alone? Our brains are faced with an overwhelming number of scenarios in everyday life that demand effective processing of complex information to shape our actions and responses. To interact with the world, our brains transfer information across sensory modalities to achieve an efficient performance. The ability to perceive and categorize objects through different sensory modalities demonstrates a complex interaction that shapes how we recognize our environment.

Category learning is an essential cognitive ability for navigating and identifying the complex surrounding environment to interact with them. This process does not rely on a single isolated brain region but involves multiple brain networks based on the task demands^1^. Category learning provides a rich framework for studying brain mechanisms involved in cross-modal information integration and related brain plasticity, as it requires combining top-down, task-driven goals with bottom-up, sensory-driven information^2^. Humans perceive the world through both unimodal and multimodal perception. Unimodal perception involves recognizing objects using a single sense, such as touch or vision. Studies have shown that visual and haptic perception of shapes are not only consistent with each other but also closely linked. For example, when a person’s visual field is restricted, their ability to visually distinguish shapes declines to a level similar to what is typically achieved through touch alone^3–6^. However, multisensory perception enables the brain to integrate information from different sensory modalities to mediate efficient behavior. Brain regions such as the lateral occipital complex (LOC) and the anterior cerebellum are critical multisensory hubs for combining visual and haptic information to facilitate identification and recognition of objects around us during daily interactions^7,8^. Another fascinating aspect of multisensory integration is cross-modal transfer of information between sensory modalities. For example, our brain applies spatial information acquired visually to haptic recognition to facilitate object identification in the absence of visual inputs. The key regions which play an important role in this process are the LOC, intraparietal sulcus (IPS), and insula/claustrum. These areas are activated during both visual and haptic object recognition^9^.

Despite these insights, prior research faces several limitations. First, studies often use familiar objects, but human perception of these is not solely driven by physical characteristics. High-level cognitive processes, such as memory and prior knowledge, significantly shape sensory integration^10–14^. Second, research employing parametric shape models, like shell-shaped 3D objects^3,15–17^, struggles to capture the complexity of natural shapes, risking confounds or oversimplification^5,18^. Third, using 2D stimuli instead of 3D avoids interactions between touch and vision, as active visual exploration often incorporates haptic cues about size and texture^19^. However, this approach does not reflect real-life scenarios, where multimodal sensory interactions are integral to object perception. To address the gap between overly familiar and entirely novel 3D objects, we utilized a virtual phylogenesis (VP) algorithm to simulate biological processes, generating a unique set of naturalistic 3D objects, termed “digital embryos”^20^. To mimic real-world conditions, we employed virtual reality to create a test environment that mirrors natural settings while minimizing haptic input during visual exploration, thus reducing experimenter influence and haptic interference^21,22^.

To our knowledge, no comprehensive dataset simultaneously explores visual and haptic category learning with unfamiliar objects to investigate cross-modal information transfer in a virtual reality to simulate real-world conditions. These gaps inspired us to develop a multimodal dataset by collecting BOLD and resting-state fMRI signals at key intervals during novel category learning experiments. We recorded resting-state fMRI data before training, immediately after category learning, 24 hours post-training, and right after the test session. We also recorded BOLD signals during unimodal (Visual-Visual, Haptic-Haptic) and cross-modal (Visual-Haptic, Haptic-Visual) test sessions for both visual and haptic sensory systems. To ensure the experiment’s interpretability, we used comparable objects for the haptic and visual tasks.

This dataset’s potential lies in its ability to probe neural plasticity associated with new category learning through resting-state and task-based brain networks, as well as applying multivariate pattern analysis (MVPA) to decode cross-modal transfer of information between sensory modalities.

While subsets of this dataset have been utilized in previous hypothesis-driven publications, the present manuscript constitutes the first comprehensive release of the complete multimodal dataset. Tabrik *et al*.^6^ analyzed exclusively the behavioral similarity rating data from the Similarity Rating Task (SRT), whereas Tabrik *et al*.^23^ and Baghernezhad *et al*.^24^ reported resting-state fMRI data collected during the first training day to investigate neural plasticity. Importantly, the behavioral data collected on the second experimental day, capturing cross-modal transfer of category knowledge during fMRI acquisition, has not been previously published. The present dataset therefore offers a novel and comprehensive resource that extends substantially beyond what has been reported in prior work.

The following sections outline the experiment designs, data acquisition equipment, protocols (Methods), the dataset’s organization (Data Records), and the technical validation of the dataset (Technical Validation).

## Methods

### Participants

The study was approved by the local ethics committee of the Medical Faculty at Ruhr-University Bochum (No. 17-6184). To recruit participants, a flyer endorsed by the Psychology Department of Ruhr-University Bochum was distributed through the campus network and student social media platforms. The study involved a total of 146 participants. All participants were right-handed as assessed by the Edinburgh Handedness Inventory test (laterality index ≥ 0.83)^25^. Of these, 50 took part in a similarity rating task (SRT). For the SRT, participants were randomly assigned to either a visual or haptic similarity rating experiment. Twenty-five participants (13 female, mean age = 23.7 ± 2.6 years) completed the visual similarity rating task, while the remaining 25 (13 female, mean age = 24.9 ± 4.6 years) participated in the haptic similarity rating task. None of the participants had prior knowledge of the object categories. For the category learning experiment, 96 participants were initially enrolled. All participants were screened to ensure they met fMRI safety criteria, excluding those with metallic implants, pregnancy, or claustrophobia. Participants reported no history of neurological conditions, no significant hand injuries (past or present), normal or corrected-to-normal vision, normal color vision, and intact hearing. Each provided written informed consent prior to the experiment and was unaware of the study’s specific objectives. Participants consented to have their data maintained, anonymized, and distributed for future research. Participants received €50 as compensation for their time. To avoid influencing resting-state connectivity patterns, study instructions were provided only after the initial resting-state fMRI data were collected^26^. Additionally, participants were not told in advance whether the second day’s testing would involve the trained or untrained sensory modality. Each participant provided written informed consent for participation and data sharing. However, data from 12 participants were excluded due to technical issues during data collection (n = 5), absence on the second day (n = 4), or failure to achieve at least 70% accuracy during training sessions (n = 3). Consequently, behavioral, resting-state, and task-based fMRI data from 84 participants were analyzed in two bimodal categorization experiments (Visual-Haptic [VH, n = 22, 11 female, mean age = 25.55 ± 3.36 years], Haptic-Visual [HV, n = 21, 10 female, mean age = 24.33 ± 4.23 years]) and two unimodal categorization experiments (Visual-Visual [VV, n = 19, 11 female, mean age = 26.26 ± 4.76 years], Haptic-Haptic [HH, n = 22, 10 female, mean age = 26.45 ± 3.93 years]).

## Experimental Design

### Stimulus generation

To incorporate realistic object properties and prevent associations with memories of familiar objects that might influence perceptual processing, we created novel naturalistic 3D objects, named digital embryos, using a Virtual Phylogenesis (VP) algorithm^27,28^. This algorithm produces a unique set of 3D object categories by simulating embryogenesis, mimicking biological processes such as cell division, growth, and movement. Starting from a uniform icosahedron as the foundational shape, the VP algorithm iteratively modifies the structure to create diverse, naturalistic forms (for further details, see http://hegde.us/digital-embryos/).

**Fig. 1:**
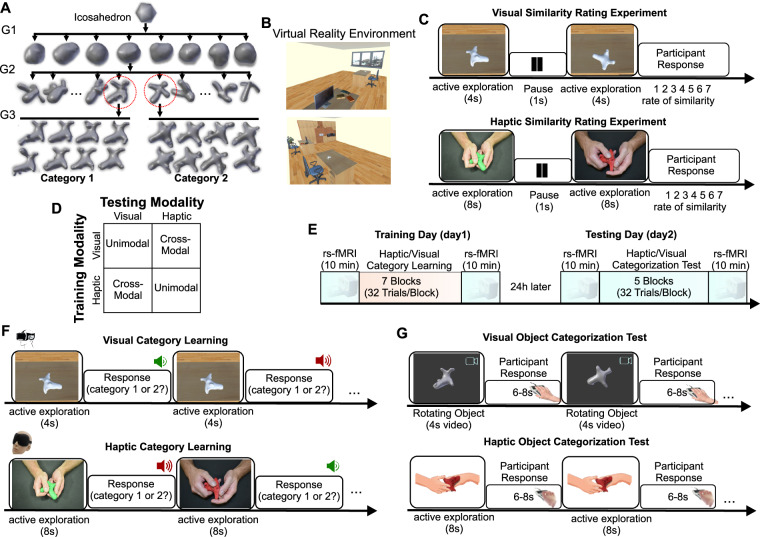
Stimuli Generation and Experimental Design. (**A**) Generation of Novel 3D Objects: Novel naturalistic 3D objects, termed digital embryos, were created using a Virtual Phylogenesis (VP) algorithm, starting from a uniform icosahedron. At each generation (Gn), selected embryos were used to produce the next generation (Gn + 1). Simulated embryonic processes—cell division, growth, and movement—were applied to a parent object from the second generation (G2, circles) to generate two distinct object categories in the third generation (G3). Each category comprised eight G3-sibling objects derived from a single parent, with siblings numbered 1–8 by the experimenter for clarity. In total, 16 objects from these two G3 categories served as stimuli for the study. Participants were unaware of the generation process or categorization criteria for these digital embryos. (**B**) Virtual Reality Environment: The experimental setting featured a virtual office furnished with a desk positioned in front of the participants. To the left, participants could see bookshelves, a printer, books, and a monitor on a study table; to the right, a window offered a view of the outdoors, enhancing the naturalistic context of the experiment. (**C**) Similarity Rating Task: A visual similarity rating task was conducted using virtual reality for the visual modality, while 3D-printed objects were used for the Haptic modality. The stimuli were identical across both modalities to ensure consistency. (**D**) Category Learning Experiments: The study included both unimodal (visual-visual, Haptic-Haptic) and cross-modal (visual-Haptic, Haptic-visual) category learning experiments to investigate sensory integration and transfer. (**E**) Experimental Design Overview: Novel category learning training was conducted outside the fMRI scanner, followed by object categorization tests performed inside the scanner to measure neural activity during task performance. Resting-state fMRI data were collected at four time points: on the first day, before and immediately after the category learning training session; and on the second day, 24 hours later, before and after the object categorization test session. Color coding indicates the recording environment: green represents sessions conducted inside the MRI scanner, and red represents sessions conducted outside the scanner. (**F**) Stimuli Presentation in Training (Day 1): This panel depicts the sequence and timing of stimuli presentation during visual and Haptic category learning experiments, highlighting modality-specific delivery protocols. Speaker icons indicate auditory feedback delivered during training: green represents feedback following a correct response and red represents feedback following an incorrect response. (**G**) Stimuli Presentation in Testing (Day 2): This panel illustrates the stimuli presentation during object categorization tests on the second day inside the fMRI scanner, using visual or Haptic modalities to evaluate learned categories in both unimodal and cross-modal conditions.

In the current study, we selected 16 embryos from two distinct categories (eight objects per category) of the third generation, as shown in Fig. 1A. As these objects had not been previously used in any object categorization experiment, we conducted similarity rating tasks in both visual and haptic sensory modalities to confirm that the new objects were perceptually distinguishable as belonging to two different categories (for more details, see Tabrik *et al*.)^6^. To this end, the key objective of the study was to identify shape features that informed similarity judgments across both visual and haptic modalities, while keeping non-shape features—such as weight, color, size, and material—constant across all objects.

### Experimental procedures

Before participating in any experiments, participants completed a brief informational survey and provided written informed consent. This study consisted of three main components: (1) a similarity rating experiment using visual and haptic modalities, (2) an fMRI study to examine unimodal and cross-modal object categorization, and (3) resting-state fMRI (rs-fMRI) to investigate neural plasticity associated with category learning and cross-modal information transfer. The similarity rating experiment was conducted to confirm that generated novel 3D objects (digital embryos) could be perceived as belonging to two distinct categories through both visual and haptic modalities^6^. Following this validation, these objects were used in subsequent category learning and object categorization experiments. The object categorization experiment spanned two consecutive days. On the first day, participants underwent category learning training in either the visual or haptic modality outside the fMRI scanner. On the second day, they were tested inside the scanner using the same training objects and novel objects in either the same (unimodal) or opposite (cross-modal) modality. To explore how category learning and cross-modal information transfer induce brain plasticity^23^, we collected resting-state fMRI data at four time points: on the first day, before and immediately after the training session, and on the second day, 24 hours later, before and after the test session. Detailed task instructions were withheld until after the first resting-state scan to minimize task-anticipatory cognition at baseline^26^. Participants nonetheless signed a full informed consent form prior to scanning, outlining general study procedures without specific task details. Longitudinal resting-state comparisons should therefore be interpreted as reflecting within-subject changes due to learning and experience over time, rather than assuming equivalent cognitive states across sessions.

### Similarity rating experiment

A standardized similarity rating task, utilizing a seven-point Likert-type scale, was developed for both visual and haptic modalities. Participants were presented with pairs of objects and asked to rate their similarity on a scale ranging from 1 (entirely dissimilar) to 7 (identical). As no explicit definition of similarity was provided, participants were free to determine the feature(s) they used for their ratings. Additionally, they were not informed that the objects belonged to two distinct categories. A total of 136 object pairs [(16 × 15)/2 + 16] were presented in random order. Drawing on a pilot study and prior research indicating that participants’ responses remain consistent across repeated stimulus presentations^3^, each pair was shown only once in the main experiment. Given that haptic exploration requires more time than visual exploration, as evidenced by prior studies showing haptic processing takes approximately twice as long^3,29,30^, we allocated 4 seconds for visual exploration and 8 seconds for haptic exploration.

In the visual similarity rating task, 16 digital embryos were displayed within a 3D virtual reality environment, allowing participants to explore the objects from all angles without physical contact. The digital embryos were imported into the Unity game engine (version 2017.2.0b8, Unity Technologies, USA) to create an immersive 3D environment for the similarity rating task. All virtual stimuli were rendered with uniform white matte materials to standardize visual appearance. The virtual environment was displayed via an HTC Vive headset (HTC and Valve Corporation) with a resolution of 2160 × 1200 pixels, a 110° field of view, and a 90 Hz refresh rate. Participants used a Vive controller to grasp and rotate objects, presented in random orientations on a virtual desk. Participants familiarized themselves with the virtual office and examined all 16 embryos (8 seconds each) before completing ten practice trials, excluded from analysis. In each trial (Fig. 1C), object pairs were shown for 4 seconds each, separated by a 1-second delay, followed by a verbal similarity rating.

For the haptic task, blindfolded participants, wearing eye masks, sat at a table with a sound-absorbing surface to ensure haptic-only exploration. They familiarized themselves with the 16-object set, exploring each for 12 seconds, followed by ten practice trials excluded from analysis. Participants freely palpated objects with both hands. In each trial (Fig. 1C), an object was placed in their hands, and a 5 kHz, 300 ms beep signalled an 8-second exploration period. A second beep prompted returning the object to the table, and the experimenter provided a second object for another 8-second exploration. Participants then verbally rated the pair’s similarity. Physical models of the same 16 objects were produced using a 3D printer (Replicator 2X, MakerBot Industries, LLC, Brooklyn, NY, USA). These hard plastic models were sanded to ensure smooth, consistent textures, eliminating surface imperfections and providing a uniform haptic experience.

In both the visual and haptic similarity rating experiments, participants were not time-restricted in reporting their similarity ratings. They were instructed to utilize the full seven-point Likert-type scale and to focus on object features when making their judgments. Verbal responses for each object pair were recorded by the experimenter. Participants could take up to three optional breaks during both experiments to minimize fatigue. The visual experiment lasted approximately 1 hour, while the haptic experiment took about 1.5 hours, reflecting the longer exploration time required for haptic processing. Following the task, participants completed a survey to identify the object features that influenced their similarity ratings. The survey listed features including (i) global shape, (ii) branch patterns, (iii) number of branches, (iv) branch size, (v) overall pattern, (vi) concavity and convexity (curvature), (vii) texture, (viii) material, (ix) color, and (x) weight. Note that texture, material, and color were held constant across all objects. These items were retained in the haptic questionnaire to maintain cross-modal comparability with the visual condition, rather than because they were expected to vary.

### Novel category learning experiment

The task-based fMRI experiment involved four groups of naïve participants: two cross-modal (Visual-Haptic, Haptic-Visual) and two unimodal (Visual-Visual, Haptic-Haptic), with 84 participants in total (see participant details in prior sections) (Fig. 1D). The experiment spanned two consecutive days: category learning training on Day 1 (outside the fMRI scanner) and testing on Day 2 (inside the scanner) (Fig. 1E). In cross-modal groups, participants trained in one modality (visual or haptic) and tested in the opposite (e.g., Visual-Haptic: visual training, haptic testing; Haptic-Visual: haptic training, visual testing). In unimodal groups, training and testing used the same modality (Visual-Visual: visual; Haptic-Haptic: haptic). Training involved 7 runs of 32 trials each (8 randomly selected “primed” objects—Category 1: obj1, obj2, obj3, obj7; Category 2: obj2, obj3, obj5, obj6—repeated four times per run), achieving 85% accuracy in the final three runs, as validated by a pilot study. The same set of primed objects was used for all participants to maintain consistency across groups and to ensure that any observed transfer effects reflect category-level learning rather than idiosyncratic object familiarity. Testing comprised 5 runs of 32 trials each (all 16 objects, primed and non-primed, with two repetitions per object). The split into primed (training) and non-primed (testing) objects enabled assessment of categorical knowledge transfer, focusing on abstraction rather than memory^20^. Visual training lasted ~60 minutes, haptic ~75 minutes, with two optional breaks per session. Post-session, participants completed a questionnaire on influential categorization features: (i) global shape, (ii) branch pattern, (iii) branch size, (iv) global size, (v) texture, (vi) color, and (vii) weight.

#### Visual training session

Participants in the Visual-Haptic (VH) and Visual-Visual (VV) groups were trained to categorize 8 primed objects (4 per category) visually within a 3D virtual reality environment. After familiarizing themselves with the VR setup and controller (Fig. 1B), participants explored each object for 4 seconds using a Vive controller with their right hand, with no restrictions. They then pressed a left (Category 1) or right (Category 2) mouse button with their left hand to categorize the object, with no time limit. Feedback was provided via a pleasant tone for correct responses or an unpleasant tone for incorrect ones. Objects appeared in pseudorandom order at a fixed location on a virtual table, randomly oriented (Fig. 1F).

#### Haptic training session

Participants in the Haptic-Visual (HV) and Haptic-Haptic (HH) groups were trained to categorize the same 8 primed objects (3D-printed versions) hapticly. Wearing eye masks to block visual input, they sat at a table covered with a foam-based sound-absorbing surface and freely palpated objects with both hands. Each trial began with an object placed on the table, signalled by a 5 kHz, 300 ms beep. After 8 seconds of exploration, a second beep indicated the end, and participants verbally categorized the object (1 or 2) without time restriction. Feedback used pleasant (correct) or unpleasant (incorrect) tones, as in the visual session. Objects were presented in pseudorandom order with random orientations (Fig. 1F).

All haptic sessions were administered by a single experimenter who received standardized training from the first author prior to data collection. The training emphasized consistency in object placement, trial timing, participant instruction, and prevention of unintended visual or auditory cues. In addition, a predefined procedural script was used throughout all sessions to standardize haptic delivery and participant instructions across trials and participants. Because all sessions were conducted by the same experimenter, inter-experimenter reliability assessment was not applicable.

#### Visual test session

On Day 2, participants underwent testing inside a 3 T MRI scanner (Achieva 3 T X, Philips Medical Systems, Best, Netherlands). To minimize motion artifacts, critical for fMRI voxel alignment, participants were instructed to remain still, with head movement restrained using foam pads. Due to MRI incompatibility with our specific VR hardware (HTC Vive), visual stimuli were presented as 4-s videos of rotating 3D objects rather than through immersive VR presentation. The videos were pre-generated prior to the experiment. For each video, an object began rotating from a randomly selected initial orientation and moved in one of eight possible directions (e.g., east–west, north–south, northeast–southwest), enabling viewing of object features from multiple viewpoints. These rotation parameters were fixed within each video and were not regenerated across trials or runs. The same stimulus set was presented to all participants, while the order of presentation was randomized independently for each participant. Each object completed two full rotations at a speed optimized for clear visual inspection and was rendered using a white matte material on a 300 × 300 pixel black background. After a 10 min resting-state fMRI, five behavioral runs were conducted. Each run began with a 20-second fixation cross, followed by 32 trials (382 seconds total, TR = 2 s, 191 volumes), and ended with a 10-second fixation. Trials consisted of a 4-second rotating object video, a 6- or 8-second inter-trial interval (ITI) with a central fixation point, and a response via an MR-compatible keyboard (Lumitouch, Photon Control Inc., Canada). Participants categorized objects using their right index (Category 1) or middle finger (Category 2), responding quickly and accurately post-stimulus without feedback. Stimuli were delivered via MR-compatible LCD goggles (Visuastim Digital, Resonance Technology Inc., Northridge, CA, USA) using Presentation software (Neurobehavioral Systems, Albany, CA) (Fig. 1G).

#### Haptic test session

On Day 2, similar to the visual experiment, participants were tested inside a 3 T MRI scanner and instructed to remain still to minimize motion artifacts. Foam pads restrained head movement, while arm and foot restraints, along with a cushion on their stomachs, supported comfortable object exploration. Participants positioned their palms on the cushion, leaving space for the experimenter to place objects without hand contact, and moved only their wrists and fingers to palpate objects. Responses were collected via two MR-compatible keyboards (Lumitouch, Photon Control Inc., Canada) under the left (Category 1) and right (Category 2) soles, using toe taps.

Five behavioral runs, each with 32 trials (16 objects, presented twice in pseudorandom order), were conducted after 10 min of resting-state fMRI. Each run began with a 20-second “Stop” fixation cue, followed by trials consisting of an 8-second object exploration period (cued by a “Play” signal), a 6- or 8-second inter-trial interval (ITI) with a central fixation point, and a toe-tap response post-“Stop” cue, without feedback. Runs ended with a 10-second fixation, totaling 510 seconds (TR = 2 s, 255 volumes). MR-compatible LCD goggles (Visuastim Digital, Resonance Technology Inc., Northridge, CA, USA) displayed “Play”/“Stop” cues to guide palpation. A 500 ms auditory tone, delivered via a 30 dB noise-attenuating headset (Visuastim Digital), signaled the experimenter to replace objects in pseudorandom order (Fig. 1).

#### Response modalities summary

Visual training used left-hand mouse buttons for categorization (to separate from right-hand VR manipulation). Haptic training used verbal responses to avoid motor confounds during exploration. In-scanner visual testing used right-hand finger presses on an MR-compatible keyboard. Haptic testing used toe presses to minimize hand movement artifacts during palpation. These choices were made to optimize natural exploration while minimizing motion artifacts and potential motor confounds in the neuroimaging data.

### Resting-State fMRI acquisition

To examine neural plasticity associated with category learning and cross-modal information transfer, resting-state fMRI data were collected at four time points: pre-training, immediately post-training, 24 hours later before the test session (pre-test), and post-test. During these acquisitions, participants performed no explicit tasks, lying relaxed with eyes closed, instructed to stay awake and avoid specific thoughts. Each resting-state scan lasted 10 minutes.

### Behavioral data analysis

To evaluate behavioral performance across all groups (VH, HV, HH, VV), mean accuracy was calculated for each run. In the training phase, as participants had no time limit and responded to all trials, no trials were excluded from accuracy analysis. For test sessions, trials with no responses or reaction times exceeding 3,000 ms were marked incorrect (VH: 2.13 ± 0.67%, HV: 1.25 ± 0.48%, HH: 2.65 ± 0.72%, VV: 2.68 ± 0.72% of trials). Mean correct response rates were then computed across participants for each test run. To compare group differences in training, the average accuracy of the final three training runs was used; for testing, the average of the last four test runs was analyzed. Two-sample t-tests (n = 6, Bonferroni-corrected for multiple comparisons) were conducted to assess the main effects of group conditions.

Two-tailed paired t-tests were conducted to compare performance between the final training run and the first test run, assessing whether category knowledge transferred across unimodal and cross-modal conditions.

### Neuroimaging data acquisition and processing

Neuroimaging data were acquired using a 3 T whole-body scanner (Achieva 3 T X, Philips Medical Systems, Best, Netherlands) with a 32-channel SENSE head coil at Bergmannsheil Hospital, Bochum, Germany.

### Data acquisition

High-resolution anatomical images for EPI-distortion correction were obtained via a T1-weighted MPRAGE sequence with the following parameters: repetition time (TR) = 8.2 ms, echo time (TE) = 3.8 ms, voxel size = 1 × 1 × 1 mm³, field of view (FOV) = 240 × 240 × 220 mm³, flip angle = 90°. Task-based functional data were collected using a T2*-weighted single-shot EPI sequence (TR = 2 s, TE = 24 ms, voxel size = 2 × 2 × 3 mm³, FOV = 224 × 224 × 125 mm³, flip angle = 80°, 38 transverse slices, slice thickness = 3 mm, inter-slice gap = 0.3 mm, multiband acceleration factor = 2). rs-fMRI data were acquired pre- and post-training on Day 1 and pre- and post-test on Day 2 using an EPI sequence (TR = 2.5 s, TE = 35 ms, flip angle = 90°, 39 slices, no gap, FOV = 224 × 224 × 117 mm³, voxel size = 2 × 2 × 3 mm³).

### fMRI data preprocessing pipeline

All neuroimaging data (task-based and resting-state) were processed using a suite of tools, including FMRIB Software Library (FSL, v6.0.5.1), Analysis of Functional NeuroImages (AFNI, v20.0.09), Advanced Normalization Tools (ANTs), and FreeSurfer (v7.1.1, Harvard University, Boston, MA, USA).

#### Task-based fMRI

DICOM files were initially converted to NIfTI format using *dcm2niix*. For fMRI time series, data quality was assessed by calculating framewise displacement (FD) using FSL’s *fsl_motion_outliers*, based on derivatives of rigid-body realignment estimates. Table 1 lists the data of participants affected by motion. These participants were excluded from further analysis. After session-specific exclusions (Table 1), the final analyzable sample per group was: VV (n = 19, 11 females, mean age = 26.26 ± 4.76), HH (n = 22, 10 females, mean age = 26.45 ± 3.93), VH (n = 22, 11 females, mean age = 25.55 ± 3.36), HV (n = 21, 10 females, mean age = 24.33 ± 4.23). Excluded sessions were not used in group analyses, but full data are available for users. In all participants, volumes where the FD exceeded 0.9 mm were modeled as confound regressors in the general linear model (GLM) analysis. Subsequently, skull and non-brain tissue were removed from anatomical images using FreeSurfer’s *recon-all* function. Motion correction was then performed to reduce motion-related artifacts using FSL’s *MCFLIRT*^31^. Non-brain tissue was removed from all fMRI data, followed by spatial smoothing with a 5 mm FWHM Gaussian filter using AFNI’s *3dBlurInMask*. For group analysis, global intensity was normalized to a grand mean of 10,000 across sessions. After standard preprocessing steps, an additional cleaning step removed in-scanner head motion artifacts using the ICA-AROMA software package (version 0.3beta). Temporal high-pass filtering (>0.01 Hz) was applied post-ICA-AROMA cleaning, as ICA benefits from the full frequency spectrum for signal separation. Slice timing correction was not applied, as the simultaneous multi-slice (multiband) acquisition excites multiple slices concurrently within each TR, violating the timing assumptions of standard slice timing correction algorithms. This approach is consistent with recommendations from the Human Connectome Project preprocessing pipeline and FSL guidelines for multiband data^32^. The first and last five volumes were discarded to ensure steady-state data and minimize filtering artifacts. EPI images were registered to high-resolution structural (MPRAGE) images using FSL’s boundary-based registration (BBR) technique (REF). Structural images were registered to the MNI152_T1_2mm template using the nonlinear Symmetric Normalization (SyN) algorithm implemented in Advanced Normalization Tools (ANTs). The nonlinear SyN transformation and BBR transformation matrix were concatenated to represent the final results in MNI space.

**Table 1:** List of session-specific exclusions due to motion.

PARTICIPANT NAME	Number of affected Volumes	Range of FD
SUB-12_SES-02_TASK-RESTPOST	37	<2.9
SUB-30_SES-02_TASK-RESTPOST	42	<4.3
SUB-35_SES-02_TASK-RESTPOST	38	<3.9
SUB-38_SES-02_TASK-RESTPOST	26	<4.9
SUB-51_SES-02_TASK-RESTPOST	27	<5.1
SUB-56_SES-02_TASK-RESTPOST	44	<3.6
SUB-30_SES-02_TASK-VISUALCAT	>26	<3.7
SUB-51_SES-02_TASK-HAPTICCAT	>25	<4.2
SUB-56_SES-02_TASK-HAPTICCAT	>19	<3.1

A session dataset was excluded from analysis if over 15% of volumes showed framewise displacement (FD) > 0.9 mm or if motion parameters exceeded half the voxel size.

#### rs-fMRI

Data preprocessing for resting-state fMRI was similar to the task-based fMRI procedures, with the following additions: After motion correction, slice-timing correction was applied using FSL’s *slicetimer* (interleaved ascending acquisition, middle slice reference). Post-ICA-AROMA, mean white matter (WM), cerebrospinal fluid (CSF), and global signals were regressed from each voxel’s time series using FSL’s *fslregfilt*. Structural images were skull-stripped and segmented into grey matter, WM, and CSF masks using FSL’s *FAST*. To prevent overlap, WM and CSF masks were binarized at a 0.95 threshold. Finally, temporal bandpass filtering (0.01–0.1 Hz) was applied, and the first and last five volumes were discarded to ensure steady-state data and minimize filtering artifacts.

### Univariate fMRI analyses

Statistical analysis was conducted using a general linear model (GLM) implemented in FSL’s FEAT (FMRI Expert Analysis Tool). To identify task-related activity, three regressors—correct trials from both categories, motor responses, and incorrect or missed trials—were convolved with a double-gamma hemodynamic response function at the individual level. Six motion regressors and framewise displacement (FD) outliers were included to account for motion effects. Two contrasts were defined: stimuli > baseline and stimuli < baseline. For each participant, GLM results from the five runs were combined using a fixed-effects model. Group-level statistics were performed with FSL’s randomize tool (Jenkinson *et al*., 2012), employing 5,000 permutations per contrast, threshold-free cluster enhancement (TFCE), and family-wise error (FWE) correction at p < 0.05. Significant clusters of activity were reported with peak voxel coordinates in MNI space (x, y, z).

### Independent component analyses of rs-fMRI data

Independent component analysis (ICA) is a computational method for identifying coherent spatial patterns in fMRI data, including resting-state networks (RSNs) and spatially structured artifacts^33,34^. ICA analysis was carried out using FSL Melodic ICA version 3.15 on the preprocessed data of pre-learning functional scans of all four groups (TT, VH, HV, and VV). Spatial ICA using 39 independent component maps (IC maps) was applied to detect RSNs from all groups.

## Data Records

The dataset for the similarity rating task is available on Mendeley^35^. The raw neuroimaging data^36^ and preprocessed data^37^, also in BIDS format, are accessible on ReSeeD. The ICA analysis for resting-state data results are available at Zenodo^38^.

The dataset’s root directory includes metadata, such as “*dataset_description.json”*, which details the dataset’s scope, acquisition protocols, and task specifications in accordance with BIDS guidelines. The “*participants.tsv*” file lists participant information, including subject ID, group assignment (VH, HV, VV, HH), age, and sex. Task-specific JSON files provide acquisition parameters for each task, including task-“*HapticCat_bold.json*” for the Haptic Categorization task and JSON files for pre- and post-training resting-state fMRI acquisitions. The neuroimaging data are organized in “sub-*” folders, each corresponding to a participant. Each participant folder contains two session subfolders (ses-01 and ses-02), representing recordings from two separate days. Each session includes an “anat” folder, which contains T1-weighted MRI data, and a “func” folder, which contains functional MRI data and behavioral event files as TSV files. Behavioral data includes exploration times, category responses, response accuracy, and feedback for both visual and haptic object categorization tasks during training and testing sessions. For ses-01, the “func” folder includes two resting-state fMRI datasets (pre- and post-training). For ses-02, the “func” folder includes five BOLD runs from a behavioral experiment (either visual or haptic categorization) and two resting-state fMRI datasets (pre- and post-test). Associated JSON files in the func folders detail the acquisition parameters for each scan.

All preprocessed data for both resting-state and task-based fMRI, generated using the steps outlined in the Methods section, are shared^37^.

## Technical Validation

### Stimuli

**Fig. 2:**
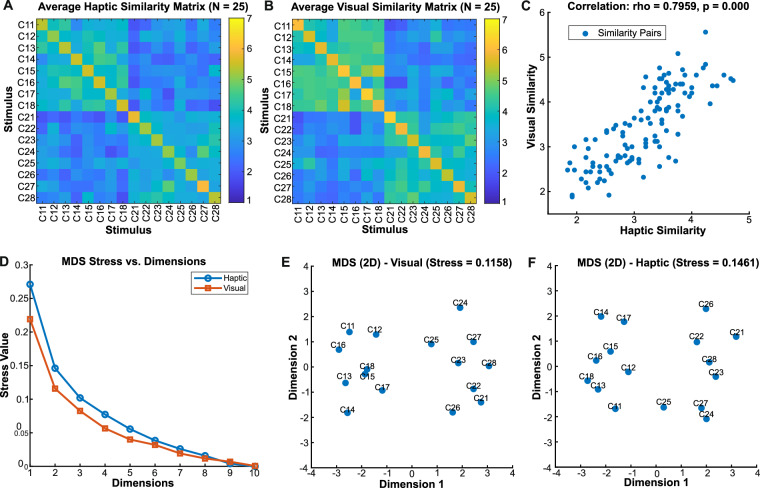
Similarity Rating Task Results. (**A**) Average similarity matrices for haptic and (**B**) visual judgments, with color-coded ratings from 1 (dissimilar, dark blue) to 7 (identical, dark red). The x- and y-axes denote the 16 digital embryos (8 per category). Cxy means object y selected from category x. (C) Correlation between haptic and visual similarity matrices. (**D**) Stress values for visual and haptic modalities across one to ten dimensions, with the elbow indicating two dimensions suffice for both perceptual spaces. (**E**) Two-dimensional perceptual spaces for visual and (**F**) haptic modalities, showing category clustering.

To ensure that the 16 digital embryos, used for the first time in a categorization experiment, were perceptually distinguished as two distinct categories, we conducted a similarity rating task in both visual and haptic modalities. Figure 2A and B display the average similarity matrices across participants, revealing a strong correlation (r = 0.8, p = 0.000) between visual and haptic similarity ratings (Fig. 2C), indicating consistent interpretation across modalities. Using average dissimilarity matrices, we performed multidimensional scaling (MDS) analysis to compute stress values across one to ten dimensions (Fig. 2D). The statistical elbow method identified two dimensions as sufficient, with stress values below 0.2, consistent with prior studies on human perceptual data^15,39,40^. The resulting perceptual spaces showed two clear clusters corresponding to the Virtual Phylogenesis (VP) algorithm’s object categories, despite participants being unaware of these categories (Fig. 2E and F).

### Behavioral data

**Fig. 3:**
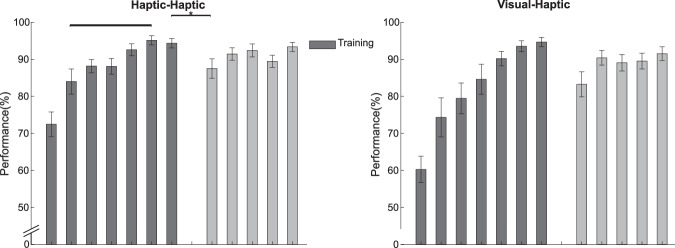
Behavioral performance of experimental groups. (**A**) performance accuracy for the HH group during training and test runs. (**B**) performance accuracy for the VH group during training and test runs. (**C**) performance accuracy for the VV group during training and test runs. (**D**) performance accuracy for the HV group during training and test runs. Each bar represents the mean ± SEM. Dark gray indicates training runs and light gray indicates test runs. “Each bar represents the mean ± SEM. An asterisk (*) indicates p < 0.01, two asterisks (**) indicate p < 0.001, and three asterisks (***) indicate p < 0.0001, all based on repeated-measures ANOVA”.

To confirm that participants learned the new categories and successfully transferred category knowledge across modalities, accuracy for each run was computed for unimodal (VV, HH) and cross-modal (VH, HV) groups, as presented in Fig. 3. Linear regression on training runs showed significant accuracy gains across all groups (HH: r² = 0.824, p = 0.0047; VH: r² = 0.917, p = 0.0007; VV: r² = 0.872, p = 0.0021; HV: r² = 0.825, p = 0.0046), confirming that all groups achieved the required learning threshold (85% accuracy) during training.

Participants trained until they maintained at least 85% accuracy over the final three consecutive runs. Mean accuracy for these runs was 94.01 ± 1.38% (mean ± SEM) for HH, 92.78 ± 1.55% for VH, 93.33 ± 1.52% for VV, and 90.16 ± 1.49% for HV. Two-sample t-tests comparing training performance across groups found no significant differences, indicating that visual and haptic training tasks were equally challenging and that all groups learned categories effectively.

Average test performance across runs was comparable: 91.65 ± 1.57% for HH, 90.12 ± 2.04% for VH, 93.07 ± 1.76% for VV, and 87.30 ± 2.54% for HV (Fig. 3). Two-sample t-tests on test performance revealed no significant group differences, suggesting that participants successfully applied learned category knowledge during testing, regardless of sensory modality.

Paired t-tests comparing the final training run to the first test run showed significant differences for VH (p = 0.005), HV (p = 0.003), and HH (p = 0.03) groups, but not for VV (p = 0.695). This suggests that cross-modal groups (VH, HV) and the haptic-only HH group required an initial test run to fully transfer category knowledge, possibly due to the novelty of haptic tasks or cross-modal recall challenges. In contrast, the VV group’s consistent performance indicates easier retention within the visual modality, likely reflecting humans’ frequent reliance on visual processing in daily life.

### Motion parameters

**Fig. 4:**
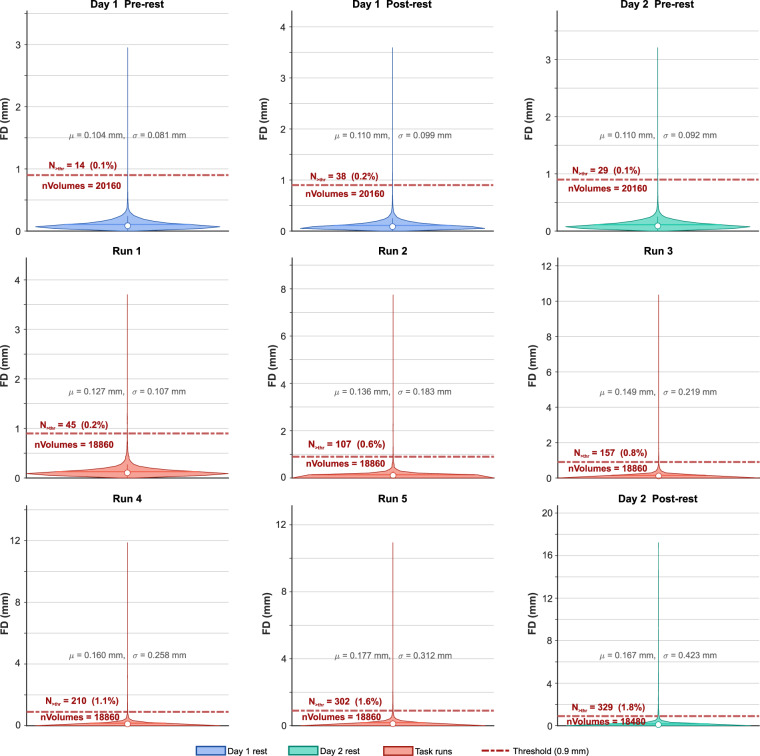
Framewise Displacement (FD) distributions across all scanning sessions. Violin plots display the distribution of framewise displacement (FD, in mm) across all volumes for each scanning session, aggregated across all participants. The top row shows resting-state sessions on Day 1 (Pre-rest: μ = 0.104 mm, σ = 0.081 mm; Post-rest: μ = 0.110 mm, σ = 0.099 mm) and Day 2 (Pre-rest: μ = 0.110 mm, σ = 0.092 mm). The middle row shows task-based fMRI runs 1–3 (Run 1: μ = 0.127 mm, σ = 0.107 mm; Run 2: μ = 0.136 mm, σ = 0.183 mm; Run 3: μ = 0.149 mm, σ = 0.219 mm). The bottom row shows task-based fMRI runs 4–5 (Run 4: μ = 0.160 mm, σ = 0.258 mm; Run 5: μ = 0.177 mm, σ = 0.312 mm) and the Day 2 Post-rest session (μ = 0.167 mm, σ = 0.423 mm). Blue violins represent Day 1 resting-state sessions, green violins represent Day 2 resting-state sessions, and red violins represent task-based fMRI runs. The horizontal dashed red line indicates the FD threshold of 0.9 mm, above which volumes were flagged as motion-contaminated. For each session, the number and percentage of volumes exceeding this threshold (N > thr) and the total number of volumes (nVolumes) are reported. The white circle within each violin indicates the median FD value. FD values generally remained well below the 0.9 mm threshold across all sessions, with the proportion of flagged volumes ranging from 0.1% in resting-state sessions to 1.8% in the Day 2 Post-rest session, confirming acceptable head motion levels across the dataset.

**Fig. 5:**
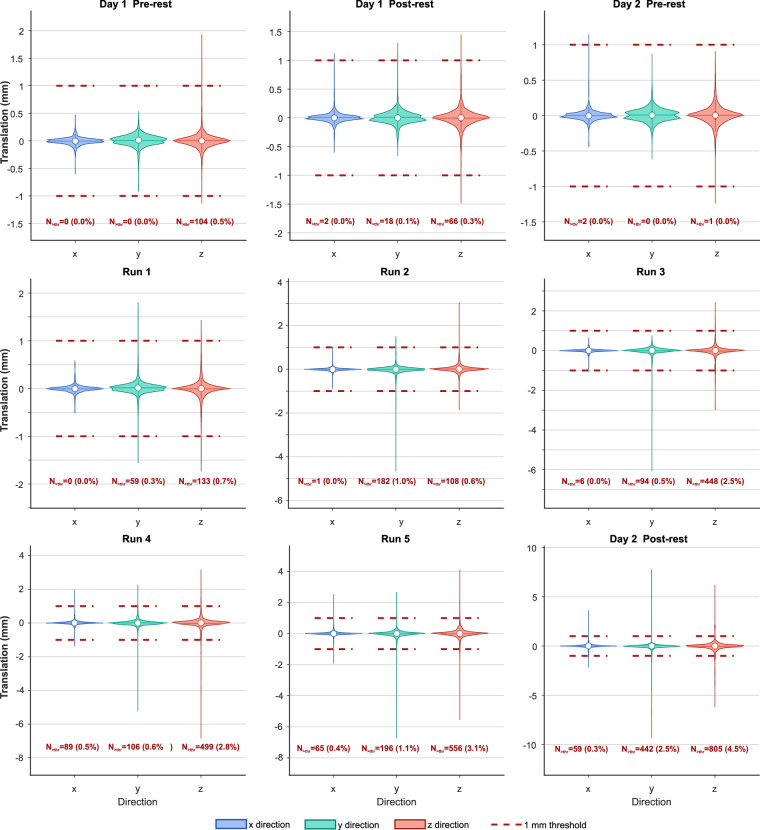
Head translation parameters across all scanning sessions. Violin plots display the distribution of head translation parameters (in mm) along the x (left-right), y (anterior-posterior), and z (superior-inferior) directions across all volumes for each scanning session, aggregated across all participants. The top row shows resting-state sessions on Day 1 (Pre-rest and Post-rest) and Day 2 (Pre-rest). The middle row shows task-based fMRI runs 1–3, and the bottom row shows task-based fMRI runs 4–5 and the Day 2 Post-rest session. Blue, green, and red violins represent translations in the x, y, and z directions, respectively. The horizontal dashed red lines indicate the ±1 mm threshold, above or below which volumes were flagged as exhibiting excessive translation. For each session and direction, the number and percentage of volumes exceeding this threshold (N > thr) are reported. Translation parameters were generally well centered around zero across all sessions and directions. The z-direction (superior-inferior) consistently showed the largest proportion of volumes exceeding the ±1 mm threshold, increasing progressively across task runs from 0.5% in Run 1 to 3.1% in Run 5, and reaching 4.5% in the Day 2 Post-rest session, reflecting cumulative head drift over the course of the scanning session. Translations in the x and y directions remained well within acceptable limits across all sessions, confirming that head motion was predominantly along the z-axis and remained at acceptable levels throughout the dataset.

**Fig. 6:**
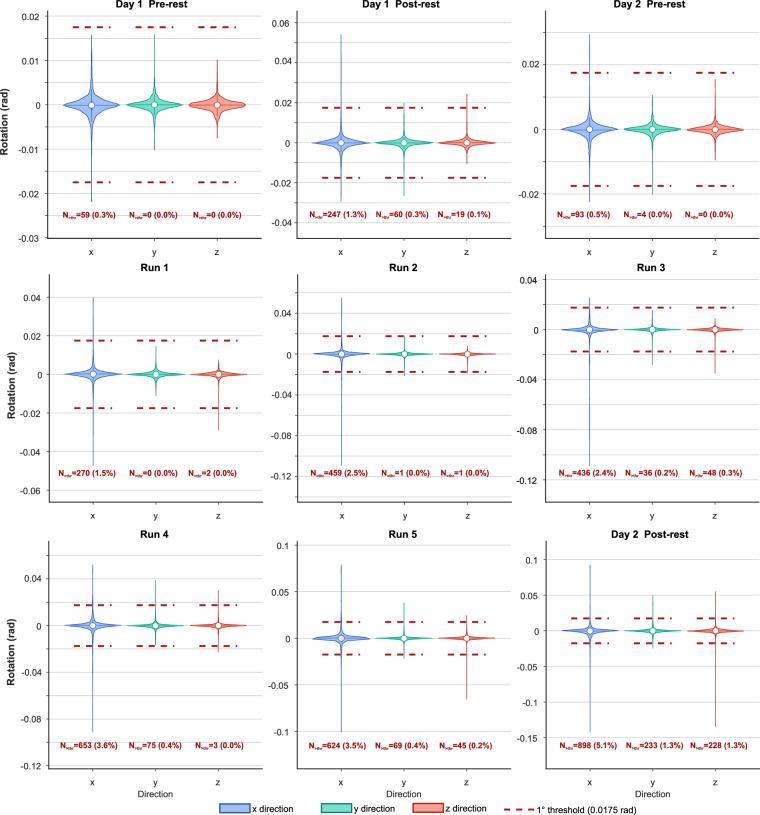
Head rotation parameters across all scanning sessions. Violin plots display the distribution of head rotation parameters (in radians) around the x (pitch), y (roll), and z (yaw) axes across all volumes for each scanning session, aggregated across all participants. The top row shows resting-state sessions on Day 1 (Pre-rest and Post-rest) and Day 2 (Pre-rest). The middle row shows task-based fMRI runs 1–3, and the bottom row shows task-based fMRI runs 4–5 and the Day 2 Post-rest session. Blue, green, and red violins represent rotations around the x, y, and z axes, respectively. The horizontal dashed red lines indicate the ±0.0175 rad (equivalent to ±1°) threshold, above or below which volumes were flagged as exhibiting excessive rotation. For each session and direction, the number and percentage of volumes exceeding this threshold (N > thr) are reported. Rotation parameters were generally well centered around zero across all sessions. Rotation around the x-axis (pitch) consistently showed the largest proportion of volumes exceeding the ±0.0175 rad threshold, increasing progressively across task runs from 1.5% in Run 1 to 3.6% in Run 4 and 3.5% in Run 5, and reaching 5.1% in the Day 2 Post-rest session. Rotations around the y and z axes remained well within acceptable limits across most sessions, with only the Day 2 Post-rest session showing slightly elevated proportions of 1.3% for both axes. The progressive increase in pitch rotation across task runs likely reflects gradual head drift during the longer Day 2 scanning session, particularly during haptic object exploration which required subtle upper body movement. Overall, rotation parameters remained at acceptable levels throughout the dataset.

To assess head motion quality across all scanning sessions, framewise displacement (FD), head translation, and rotation parameters were computed for all participants across all sessions using FSL’s *fsl_motion_outliers*. FD distributions remained well below the 0.9 mm threshold across all sessions, with the proportion of flagged volumes ranging from 0.1% in resting-state sessions to 1.8% in the Day 2 Post-rest session (Fig. 4). Translation parameters were generally centered around zero across all sessions, with the z-direction (superior-inferior) showing the largest proportion of volumes exceeding the ± 1 mm threshold, increasing progressively from 0.5% in Run 1 to 4.5% in the Day 2 Post-rest session (Fig. 5). Rotation parameters similarly remained within acceptable limits, with pitch rotation (x-axis) showing the largest exceedances, reaching a maximum of 5.1% in the Day 2 Post-rest session, likely reflecting gradual head drift during the longer haptic scanning session (Fig. 6). Volumes exceeding motion thresholds were modeled as confound regressors in the GLM, and sessions where more than 15% of volumes exceeded the FD threshold of 0.9 mm were excluded from further analysis (Table 1). These results confirm that head motion remained at acceptable levels throughout the dataset.

### General activation maps

**Fig. 7:**
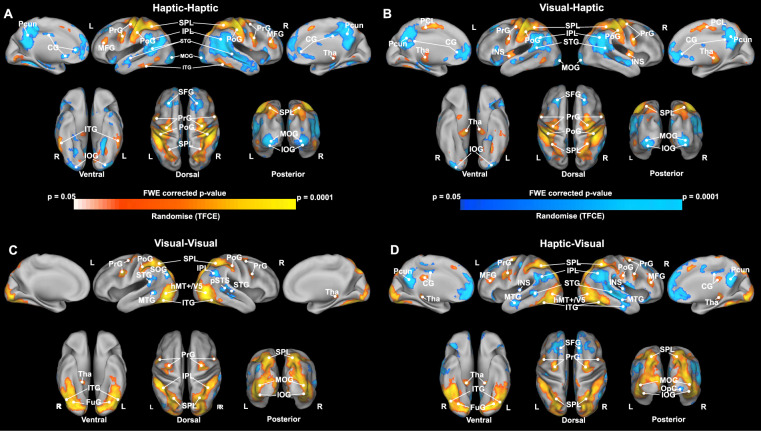
Brain Activation and Deactivation Patterns Across Groups. (**A**) Haptic object categorization in the HH group. (**B**) Haptic object categorization in the VH group. (**C**) Visual object categorization in the VV group. (**D**) Visual object categorization in the HV group. Significantly increased BOLD signals are depicted in red-to-yellow colors, while significantly decreased BOLD signals are shown in blue. Statistical analyses were performed using FSL’s Randomise nonparametric permutation testing tool (Jenkinson *et al*., 2012) with 5000 permutations per contrast, threshold-free cluster enhancement (TFCE), and family-wise error (FWE) correction, thresholded at p < 0.05.

To evaluate the quality of task-based fMRI data, we preprocessed the data and conducted GLM analysis to identify task-positive (categorization > baseline) and task-negative (categorization < baseline) activation patterns. The neuroimaging findings revealed widespread task-positive brain activity during visual and haptic categorization tasks across the VV, HV, VH, and HH groups (Fig. 7). The activated regions are summarized in Table 2. These findings highlight modality-specific and cross-modal neural processing during visual and haptic object categorization, with significant activations observed in sensory, motor, associative cortical, and subcortical regions. The HH group, trained and tested in the haptic modality, showed robust activations in the bilateral postcentral gyri, the primary somatosensory cortex (S1), bilateral cerebellar regions, small clusters in the right superior frontal gyrus, bilateral middle frontal gyri, right thalamus, and left insula. The VH group, trained visually but tested hapticly, exhibited the largest activation cluster in the right postcentral gyrus, followed by the left postcentral gyrus, bilateral cerebellar regions, bilateral thalamus, right lentiform nucleus, and left paracentral lobule. The VV group, trained and tested in the visual modality, showed prominent activations in the bilateral lateral occipital cortex (V4, LOC), bilateral precentral gyri, and smaller clusters in the right thalamus and left lentiform nucleus. The HV group, trained hapticly but tested visually, displayed significant activations in the bilateral temporal occipital fusiform cortex, bilateral precentral gyri, bilateral middle frontal gyri, right superior frontal gyrus, and right thalamus.

**Table 2:** Brain Regions Exhibiting Task-Positive Activation During Categorization.

Brain Area		Cluster volume(mm^3^)	MNI coordinate			Z-value
			x	y	z	
Haptic-Haptic	L Postcentral Gyrus	7983	−38	−24	52	6.26
	R Postcentral Gyrus	7651	46	−24	56	6.13
	L Precentral Gyrus	382	−49	5	34	5.25
	R Precentral Gyrus	592	33	−11	51	5.11
	L Cerebellum	3584	−26	−56	−22	6.18
	R Cerebellum	3423	20	−56	−27	5.95
	R Middle Frontal Gyrus	477	42	36	22	4.76
	L Middle Frontal Gyrus	256	−42	32	20	4.19
	L Insula	231	−40	−6	14	4.71
	R Superior Frontal Gyrus	184	6	14	48	4.75
	R Thalamus (Pulvinar and VPL)	152	16	−24	6	4.58
	L Superior Parietal Lobule	358	−22	−62	58	5.75
	R Superior Parietal Lobule	388	16	−66	52	4.95
	L Inferior Temporal Gyrus	412	−48	−46	−22	5.35
	R Inferior Temporal Gyrus	357	54	−50	−18	4.96
Visual-Haptic	L Postcentral Gyrus	5956	−40	−26	50	6.98
	R Postcentral Gyrus	72933	40	−28	50	6.79
	R Cerebellum	3025	30	−56	−24	6.79
	L Cerebellum	2985	−26	−56	−24	6.52
	L Paracentral Lobule	978	−6	−24	50	4.87
	R Paracentral Lobule	792	10	−21	53	5.11
	L Thalamus (Pulvinar and VPL)	918	−16	−22	8	5.30
	R Thalamus (Pulvinar and VPL)	479	16	−22	0	5.19
	R Lentiform Nucleus (Lat Glob Pallidus)	622	20	0	2	5.51
	L Superior Parietal Lobule	410	−19	−59	60	4.58
	R Superior Parietal Lobule	375	16	−63	55	4.86
	L Insula	176	−22	24	−7	4.45
	R Insula	168	33	13	2	4.68
	L Postcentral Gyrus	6973	−40	−24	56	5.86
	R Postcentral Gyrus	6215	45	−24	52	8.53
	L Precentral Gyrus	412	−33	10	35	4.85
	R Precentral Gyrus	576	49	−6	49	5.23
Visual-Visual	L Lateral Occipital Cortex	10123	−42	−66	−4	6.20
	R Lateral Occipital Cortex	11201	44	−72	−4	5.95
	L Precentral Gyrus	482	−46	5	33	5.28
	R Precentral Gyrus	392	40	−11	49	4.94
	L Postcentral Gyrus	7856	−46	−21	50	6.52
	R Postcentral Gyrus	7215	40	−29	52	7.93
	R Thalamus (Pulvinar)	122	20	−30	0	4.42
	L Lentiform Nucleus (Putamen)	68	−26	−2	12	4.00
	L hMT + /V5	121	−46	−72	5	4.67
	R hMT + /V5	98	43	−70	8	4.44
	L Inferior Temporal Gyrus	436	−61	−36	−12	5.75
	R Inferior Temporal Gyrus	389	50	−50	−9	4.96
	L Fusiform Gyrus	14025	−38	−47	−14	5.97
	R Fusiform Gyrus	8254	44	−59	−14	5.23
	L Superior Parietal Lobule	358	−16	−70	52	5.31
	R Superior Parietal Lobule	342	19	−66	58	4.93
	L Inferior Parietal Lobule	453	−42	−44	53	5.18
	R Inferior Parietal Lobule	398	36	−41	49	4.87
Haptic-Visual	R Fusiform Gyrus	14025	42	−48	−14	6.27
	L Fusiform Gyrus	8254	−46	−62	−14	5.42
	L Middle Frontal Gyrus	325	−28	34	34	4.47
	R Middle Frontal Gyrus	845	30	−2	62	5.18
	L Precentral Gyrus	752	−48	4	34	4.50
	R Precentral Gyrus	627	24	−16	54	5.31
	R Middle Frontal Gyrus	407	50	42	12	4.67
	L Thalamus (Pulvinar)	369	−23	−32	2	4.56
	R Thalamus (Pulvinar)	389	24	−32	0	4.72
	L Postcentral Gyrus	324	−46	−16	50	4.52
	R Postcentral Gyrus	221	56	6	28	3.93
	L Middle Occipital Gyrus	893	−31	−92	−2	5.62
	R Middle Occipital Gyrus	793	33	−92	−8	4.91
	L Inferior Temporal Gyrus	387	−57	−39	−11	5.15
	R Inferior Temporal Gyrus	403	57	−56	−12	5.26
	L hMT + /V5	112	−47	−70	2	4.39
	R hMT + /V5	87	44	−70	11	4.16
	L Superior Parietal Lobule	418	−22	−66	55	5.35
	R Superior Parietal Lobule	366	16	−62	51	5.45
	L Inferior Occipital Gyrus	234	−41	−76	−5	4.81
	R Inferior Occipital Gyrus	358	38	−78	12	4.37

This table lists brain regions with significant activations (increased BOLD signals) during the categorization task compared to baseline across four groups (Task > Baseline): Haptic-Haptic (HH), Visual-Haptic (VH), Visual-Visual (VV), and Haptic-Visual (HV). The data include cluster volumes (mm³), MNI coordinates (x, y, z), and Z-values, indicating the strength of activation. p < 0.05 for all reported activations.

In contrast, regions showing significantly reduced BOLD signals during the categorization task compared to baseline were observed across all groups and are summarized in Table 3. These deactivations primarily involved regions associated with the default mode network (DMN) and other task-irrelevant areas. For the HH group, deactivations included the left precuneus cortex, right superior temporal gyrus, bilateral superior frontal gyri, bilateral middle occipital gyri, and left superior temporal gyrus. The VH group showed deactivations in the right inferior parietal lobe, left supramarginal gyrus, left precuneus cortex, left medial frontal gyrus, left superior frontal gyrus, bilateral middle occipital gyri, and right superior temporal gyrus. The VV group exhibited deactivations in the left superior occipital gyrus, right middle temporal gyrus, and bilateral supramarginal gyri. The HV group displayed deactivations in the right superior temporal gyrus, left anterior cingulate, left supramarginal gyrus, left precuneus, left paracentral lobule, left middle temporal gyrus, right superior frontal gyrus, and right middle frontal gyrus. These deactivation patterns suggest suppression of internally focused or task-irrelevant processes during categorization.

**Table 3:** Brain Regions Exhibiting Task-Negative Deactivation During Categorization.

Brain Area		Cluster	MNI coordinate			Z-value
Haptic-Haptic	L Precuneus cortex	8904	−16	−38	50	5.79
	R Precuneus cortex	7890	20	−41	50	5.56
	L Superior Temporal Gyrus	469	−36	2	−18	4.32
	R Superior Temporal Gyrus	6319	56	−40	14	6.22
	L Superior Frontal Gyrus	902	−32	32	48	4.80
	R Superior Frontal Gyrus	3982	14	32	62	5.18
	L Middle Occipital Gyrus	913	−30	−92	−2	4.66
	R Middle Occipital Gyrus	856	32	−92	−8	5.01
	L Inferior Occipital Gyrus	345	−40	−73	−5	4.83
	R Inferior Occipital Gyrus	298	38	−76	12	4.44
	L Inferior Parietal Lobule	389	−40	−41	53	4.98
	R Inferior Parietal Lobule	368	37	−43	50	4.86
	L Cingulate Gyrus	232	−2	2	50	4.18
	R Cingulate Gyrus	211	2	−2	48	4.04
Visual-Haptic	L Inferior Parietal lobe	2966	−58	−50	42	4.98
	R Inferior Parietal lobe	3466	60	−48	42	5.21
	L Supramarginal Gyrus	2878	−64	−44	36	5.18
	L Precuneus cortex	2787	−8	−48	40	5.02
	R Precuneus cortex	3890	16	−43	46	5.36
	L Medial Frontal Gyrus	2104	−8	58	−2	4.70
	L Superior Frontal Gyrus	983	−40	26	48	5.15
	R Superior Frontal Gyrus	763	38	23	41	4.74
	L Middle Occipital Gyrus	635	−26	−86	−6	4.36
	R Middle Occipital Gyrus	773	32	−90	2	4.52
	L Superior Temporal Gyrus	570	−60	−4	−15	4.67
	R Superior Temporal Gyrus	660	54	−4	−10	4.31
	L Inferior Occipital Gyrus	301	−44	−71	−11	5.01
	R Inferior Occipital Gyrus	325	40	−73	8	4.76
	L Cingulate Gyrus	262	−5	1	50	3.98
	R Cingulate Gyrus	256	2	−2	48	4.12
Visual-Visual	L Superior Occipital Gyrus	935	−40	−80	40	5.48
	R Middle Temporal Gyrus	863	62	−24	−8	4.93
	R Supramarginal Gyrus	590	56	−52	38	5.22
	L Supramarginal Gyrus	177	−64	−48	40	4.47
	L Superior Temporal Gyrus	2369	−40	3	−20	4.72
	R Superior Temporal Gyrus	3345	53	−40	14	5.12
	R posterior superior temporal sulcus	523	50	−47	13	5.02
Haptic-Visual	L Superior Temporal Gyrus	4756	−60	−25	−2	5.34
	R Superior Temporal Gyrus	5217	58	−22	−6	5.56
	L Anterior Cingulate	5191	−6	40	−16	5.54
	R Anterior Cingulate	4356	9	40	−16	4.98
	L Supramarginal Gyrus	1596	−62	−48	26	4.71
	L Precuneus	1281	−2	−60	26	5.19
	R Precuneus	987	2	−57	22	5.23
	L Paracentral Lobule	606	−2	−26	46	5.73
	L Middle Temporal Gyrus	505	−64	−12	−18	4.99
	R Middle Temporal Gyrus	486	59	−16	−17	5.12
	L Superior Frontal Gyrus	259	−2	16	46	4.59
	R Superior Frontal Gyrus	379	36	30	−16	5.25
	L Inferior Parietal Lobule	351	−42	−43	53	4.98
	R Inferior Parietal Lobule	293	36	−42	51	5.17
	L Insula	189	−21	21	−6	4.15
	R Insula	178	33	18	4	4.34

This table lists brain regions with significant deactivations (decreased BOLD signals) during the categorization task compared to baseline across four groups (Task < Baseline): Haptic-Haptic (HH), Visual-Haptic (VH), Visual-Visual (VV), and Haptic-Visual (HV). The data include cluster volumes (mm³), MNI coordinates (x, y, z), and Z-values, indicating the strength of activation. p < 0.05 for all reported activations.

### Resting-state networks

**Fig. 8:**
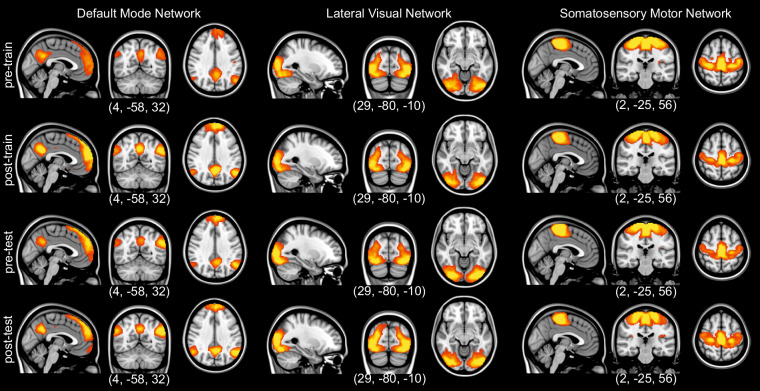
Reproducibility of resting-state networks across different sessions demonstrates data quality consistency. ICA was performed on each of the four resting-state datasets (pre-training, post-training, pre-test, post-test) to assess data quality and network reproducibility. Representative spatial maps are shown for three well-characterized resting-state networks as examples: the default mode network (top row), lateral visual network (middle row), and motor network (bottom row). Consistent spatial patterns across all four sessions indicate reliable data acquisition and preprocessing, validating the quality of the resting-state recordings for subsequent analyses. Maps are displayed at z > 4 and overlaid on a standard brain template.

To ensure the quality of the resting-state fMRI dataset, we implemented a rigorous quality control pipeline and conducted group-level Independent Component Analysis (ICA) to identify robust functional networks, including the Default Mode Network (DMN). After preprocessing (see Method section), using FSL’s MELODIC, we performed ICA on resting-state data from all 84 participants for all four sessions (ses-01 pre/post, ses-02 pre/post) separately, identifying consistent networks like the DMN (precuneus, medial prefrontal cortex), visual, and sensorimotor networks (Fig. 8).

To assess the reproducibility of the ICA results, we computed the Intraclass Correlation Coefficient (ICC(3,1); two-way mixed-effects model, consistency definition) for the spatial maps of each identified resting-state network (RSN) across the four scanning sessions (Pre-training, Post-training, Pre-test, and Post-test; N = 84). ICC(3,1) was calculated on the subject-specific spatial maps derived via dual regression, using the mean z-score within each RSN mask as the summary metric per subject per session. ICC estimates and their 95% confidence intervals (CIs) were computed using the pingouin library in Python (Vallat, 2018)^41^, and reproducibility was interpreted according to the benchmarks proposed by Koo and Li (2016)^42^: poor (<0.50), moderate (0.50–0.74), good (0.75–0.90), and excellent (>0.90). ICC(3,1) values across all identified RSNs ranged from 0.56 (95% CI [0.50–0.62]) to 0.73 (95% CI [0.63–0.83]), indicating moderate to good reproducibility of the ICA spatial maps across sessions. These findings confirm that the observed between-session differences in RSN topology reflect genuine neurophysiological changes rather than measurement instability.

The three components (DMN, LVN and SMN) were examined in detail. DMN demonstrated good reproducibility across all four sessions (ICC(3,1) = 0.730, 95% CI [0.630–0.820], *F*(76, 228) = 12.805, *p* < .001), with consistent estimates across pairwise comparisons: between-day reproducibility (Pre-Day 1 vs. Pre-Day 2) was ICC = 0.719 (95% CI [0.570–0.840]), within-Day 1 reproducibility (Pre- vs. Post-training) was ICC = 0.782 (95% CI [0.650–0.88]), and within-Day 2 reproducibility (Pre- vs. Post-test) was ICC = 0.733 (95% CI [0.580–0.850]), suggesting stable spatial expression of this network across both days and both interventions. LVN showed overall moderate reproducibility (ICC(3,1) = 0.658, 95% CI [0.550–0.760], *F*(76, 228) = 10.050, *p* < .001), with a distinctive pattern in which within-Day 2 reproducibility (ICC = 0.732, 95% CI [0.580–0.850]) exceeded both the between-day estimate (ICC = 0.651, 95% CI [0.480–0.780]) and the within-Day 1 estimate (ICC = 0.545, 95% CI [0.490–0.600]), suggesting that the spatial topology of this network was particularly consistent within the test day session. SMN showed good overall reproducibility (ICC(3,1) = 0.646, 95% CI [0.540–0.750], *F*(76, 228) = 10.818, *p* < .001), with notably balanced estimates across all pairwise comparisons: between-day ICC = 0.714 (95% CI [0.560–0.830]), within-Day 1 ICC = 0.673 (95% CI [0.510–0.800]), and within-Day 2 ICC = 0.672 (95% CI [0.500–0.810]). Taken together, these three networks shared a common feature of moderate-range between-day ICC values, consistent with the broader rs-fMRI test-retest literature, and collectively provide the strongest evidence for the spatial reliability of the ICA solution across the two-day protocol.

These findings confirm the quality and reliability of the resting-state fMRI acquisition and preprocessing, demonstrating that the data are suitable for investigations of resting-state functional connectivity and network reproducibility.

## Data Availability

The datasets described in this manuscript are openly available at the following repositories: raw neuroimaging data (10.60517/0874db71-e087-444b-96eb-41b97dc49a7d), preprocessed neuroimaging data (10.60517/80bf4025-2594-4a46-93b2-b729867d6d48), and similarity rating experiment data (https://data.mendeley.com/datasets/8k7ys2xw4h/1).

## Code availability

The stimuli and data preprocessing scripts for the similarity rating task are available on GitHub at https://github.com/Sepideh-Tabrik/Similarity-Rating-Task. The code for neuroimaging data processing is accessible at https://github.com/Sepideh-Tabrik/Neural-mechanisms-underlying-cross-modal-3D-category-learning.
